# The Role of Micromorphology of SnS_2_ on the Adsorption Process of Methylene Blue

**DOI:** 10.3390/molecules31101624

**Published:** 2026-05-12

**Authors:** Hao Guo, Wenjie Gao, Lang Yang, Zhuolin Qin, Feng Rao

**Affiliations:** 1 Zijin School of Geology and Mining, Fuzhou University, Fuzhou 350108, China; guohao716@foxmail.com (H.G.); gaowenjie0327@foxmail.com (W.G.); qinzl_edu@163.com (Z.Q.); fengrao@fzu.edu.cn (F.R.); 2 Fujian Key Laboratory of Green Extraction and High-Value Utilization of New Energy Metals, Fuzhou 350108, China

**Keywords:** SnS_2_, methylene blue, morphological difference, adsorption process

## Abstract

In this study, three SnS_2_ samples with different morphologies were synthesized using a one-step hydrothermal method, and the effect and mechanism of morphology on their adsorption ability toward methylene blue (MB) were studied. XRD and SEM revealed effective preparations of SnS_2_ with flake, flower-like, and granular morphologies, as well as their size variations. The BET results indicate that the specific surface areas follow the order flower-like > granular > flake. Adsorption experiments demonstrated that the morphology of SnS_2_ considerably impacts their MB adsorption ability. Kinetic investigations implied that the adsorption of MB on flower-like and granular SnS_2_ followed a pseudo-second-order kinetic model, with adsorption rates in the order of flower-like > granular. MB adsorption on flake SnS_2_ followed the Weber–Morris model. The adsorption of MB on all three SnS_2_ structures followed the Langmuir isotherm model, with the flower-like SnS_2_ exhibiting the highest maximum adsorption capacity of 33.1 mg/g, which is 28.8% and 27.8% higher than that of the flake structure (25.7 mg/g) and the granular structure (25.9 mg/g), respectively. Adsorption thermodynamics indicated that the ΔG^θ^ for MB adsorption on all morphologies was negative, suggesting their spontaneous adsorption process. Furthermore, ΔG^θ^ decreased with increasing temperature, indicating that higher temperatures promote MB adsorption. In addition, both the values of ΔH^θ^ and the ΔS^θ^ for the MB adsorption on SnS_2_ were in the order of flower-like > granular > flake SnS_2_, suggesting that MB is more easily adsorbed on the flower-like SnS_2_ than granular SnS_2_ and final flake SnS_2_. DFT simulations confirmed that the distinct exposed facets of the flower-like morphology yielded the strongest adsorption energies, revealing the essential structure–property relationship for designing highly efficient 2D adsorbents. This work reveals the important effect of SnS_2_ morphology on its adsorption behavior and gives essential insights for the design and development of adsorbents.

## 1. Introduction

With the rapid development of the chemical industry, dyes (e.g., MB) are widely used in textile, paper, and printing industries and generate a large amount of wastewater [1]. However, some of the untreated dye wastewater is often discharged directly or indirectly into natural water bodies for different reasons [2], leading to water pollution and ecological damage and posing a serious threat to human health [3].

Due to the stable molecular structure and difficult biodegradation of MB, its cumulative effect in the environment makes the efficient removal of MB an urgent problem in the field of water treatment [4]. Among the existing MB removal methods, adsorption has attracted much attention due to its easy operation, low cost, and good regeneration performance [5,6]. The adsorption behaviors of MB on various adsorbents, such as activated carbon [7,8], mesoporous silica [9], layered porous MgO [10], porous geopolymer [11], and iron oxide [12], have been extensively studied. The results of adsorbents in terms of adsorption capacity [13], adsorption efficiency [14], mechanical properties, and recoverability [15] indicate that material components, structural configurations, and synthesizing methods have an important influence on adsorption properties [16]. But, traditional adsorbents such as clay [17] and activated carbon [18] generally face issues of limited capacity and slow kinetics, and they also have poor selectivity for removing MB, combined with weak affinity. In contrast, 2D materials have richer active adsorption sites, which can effectively bind to pollutant molecules, resulting in rapid adsorption, which may be better for MB [19]. Nevertheless, the surface hydrophobicity of many 2D materials severely hinders their adsorption of aqueous-phase pollutants, necessitating chemical modifications to achieve effective dye removal [20], and current studies on 2D materials are mainly focused on catalysis and electrochemistry. Nano-MoS_2_ has attracted attention due to its excellent chemical, electronic, catalytic, optical, and mechanical properties, but, when used for heavy metal adsorption, its hydrophobicity and relatively low dispersion pose some challenges to adsorption performance [21]. Some studies have been conducted to improve their hydrophilicity through surface modification (e.g., introduction of sulfur vacancies or metal doping), but these methods are often accompanied by complex synthesis steps or decreased material stability [20]. Therefore, hydrophilic 2D materials are of great interest in the treatment of aqueous-phase pollutants.

SnS_2_ is a layered material with broad spectral response, good dispersion, and a large specific surface area and has been widely used in gas-sensitive devices [22], solar cells [23], and photocatalysis [24]. More importantly, the natural hydrophilic nature of SnS_2_ may give it a unique advantage in the adsorptive removal of pollutants in the aqueous phase. The Sn–S bonding characteristics of SnS_2_, along with the formation of polar sites and unsaturated bonds due to surface sulfur vacancies, endow it with significantly greater surface hydrophilicity than MoS_2_. This strong hydrophilicity has important practical implications—SnS_2_ can rapidly draw dye solutions into its interior via capillary action. Recent studies have demonstrated that the nanogully structure of SnS_2_ can overcome conventional diffusion limitations, enabling capillary-driven enrichment of dyes, a phenomenon not observed in MoS_2_ under comparable conditions. Moreover, SnS_2_ surfaces can spontaneously adsorb cationic species, whereas MoS_2_ generally requires surface functionalization to effectively bind cationic dyes. Notably, SnS_2_ can be synthesized in various morphologies, such as nanosheets, flower-like structures, and particles, by adjusting synthesis conditions. This morphological tunability enables precise control over specific surface area, pore structure, and the density of active edge sites, all of which play a critical role in determining its adsorption performance [25]. It has been shown that some SnS_2_ exhibits excellent performance in the photocatalytic degradation of MB [26,27]. Meanwhile, SnS_2_ exhibits higher catalytic degradation of MB when the SnS_2_ become thinner [28]. However, most current studies mainly focus on the photocatalytic efficiency of SnS_2_, while few studies examine the differences in pollutants on the surfaces of different morphologies of SnS_2_ before the photocatalytic reaction.

Therefore, understanding the surface interactions between varying SnS_2_ micro-architectures and aqueous pollutants prior to photocatalytic degradation is of critical significance. The main objective of this study is to systematically investigate the precise role of SnS_2_ micromorphology on its interfacial adsorption behavior toward MB. To accomplish this, three specific tasks were undertaken: (1) controlled synthesis of flake, flower-like, and granular SnS_2_ via a one-step hydrothermal method and their structural confirmation via XRD, SEM, and BET; (2) comprehensive evaluation of their adsorption properties through pH dependency, kinetic, isothermal, and thermodynamic experiments; and (3) atomic-level elucidation of the adsorption mechanisms utilizing FT-IR spectroscopy and density functional theory (DFT) calculations. By explicitly linking macroscopic adsorption capacities with specific surface areas and ab initio interaction energies of different crystal facets, this work provides empirical and theoretical insights essential for the targeted design of high-performance SnS_2_-based environmental materials.

## 2. Experiment

### 2.1. Materials and Reagents

Stannic chloride pentahydrate (SnCl_4_·5H_2_O), thiourea (CH_4_N_2_S), oleylamine (C_18_H_37_NH_2_), solvents (deionized water, ethanol), methylene blue (MB), sodium hydroxide (NaOH), and hydrochloric acid (HCl) were purchased from Sinopharm Chemical Reagent Co., Ltd. (Shanghai, China). Ultrapure water with a resistivity of 18.2 M Ω·cm (prepared using a Millipore Super Q system, Burlington, MA, USA) was used throughout the experiments. All solutions were prepared with ultrapure water as the solvent.

### 2.2. Synthesis of SnS_2_ and MB Adsorption

Flake SnS_2_: 0.4 mmol of SnCl_4_·5H_2_O and 2 mL of oleylamine were added into 37 mL of anisole at room temperature, and the mixture was heated to 70 °C and stirred for 30 min. The reaction solution was poured into a 50 mL PTFE container, 1.0 mL of CS_2_ was added, and it was placed into a steel autoclave and sealed. After heating at 180 °C for 24 h, the reaction kettle was removed, naturally cooled to 50–60 °C, centrifuged, washed repeatedly with anhydrous ethanol, and dried under vacuum [29].

Flower-like SnS_2_: SnCl_4_·5H_2_O was used as the tin source, 0.701 g was weighed, thiourea was used as the sulfur source, 0.457 g was weighed, the solvent used was deionized water, 35 mL was measured, and 20 mL of Polyethylene Glycol 400 (PEG 400) with a volume fraction of 40% was added and stirred homogeneously to obtain a clarified solution. The solution was placed in a 100 mL hydrothermal kettle liner and kept warm at 180 °C in an electrothermal thermostat for 15 h and then naturally cooled to room temperature. The sample was washed with deionized water and alcohol three times and finally dried at 60 °C for 12 h in a vacuum chamber [30].

Granular SnS_2_: 1.75 g of SnCl_4_·5H_2_O and 1.5 g of thiourea were sequentially weighed and added to 35 mL of deionized water; after stirring to make it fully mixed, the resulting solution was transferred to a stainless steel reactor with a volume of 50 mL lined with PTFE, the volume was filled to 70%, and the reaction was hydrothermal at 160 °C for 8 h. After the reaction, the reactor was cooled to room temperature, and the yellow precipitate was washed through centrifugation repeatedly and dried under vacuum at 70 °C for 5 h. The reaction was carried out in a stainless steel reactor lined with PTFE [31].

### 2.3. MB Adsorption Experiment

In a typical adsorption experiment, MB concentration 10 mg/L, MB volume 50 mL, stirring speed 600 r/min, adsorbent dosage 25 mg, pH 7, adsorption time 480 min, and 2 mL of the solution was taken at certain intervals between adsorptions, filtered through 0.22 μm filter membrane, and the concentration was tested in a spectrophotometer. The adsorption of SnS_2_ on MB was calculated according to Equation (1).

(1) $$q=\frac{(C_{0}-C_{e})\times V}{m}$$

In the equation, *q* is the adsorbed amount at adsorption equilibrium, mg/g; *C*_0_ is the initial concentration of MB solution, mg/L; *C_e_* is the concentration of MB solution at equilibrium time, mg/L; *m* is the amount of SnS_2_, g; and *V* is the volume of MB solution, L. The adsorption equilibrium is the time of adsorption equilibrium.

### 2.4. Characterization Methods

A UV-vis spectrophotometer was used to monitor the adsorption effect of the samples on MB. The crystalline structures of the samples were examined using an X-ray diffractometer (XRD, D8 Advance, Bruker, Billerica, MA, USA). The morphologies were observed with a scanning electron microscope (SEM, Ultra Plus, Zeiss, Jena, Germany). Specific surface areas and pore structures were determined by a surface area and porosity analyzer (BET, TriStar II Plus, Micromeritics, Norcross, GA, USA). The functional groups on the SnS_2_ after MB adsorption were analyzed by Fourier transform infrared spectroscopy (FTIR, [Nicolet iS50, Thermo Fisher Scientific, Waltham, MA, USA]). The adsorption of MB was monitored using a UV–vis spectrophotometer ([UV-2600, Shimadzu, Kyoto, Japan]).

### 2.5. DFT Parameter Settings

In order to study the adsorption of SnS_2_ on MB, three surface structure models of SnS_2_, flake, flower-like, and granular were constructed to calculate the adsorption energy of MB on the surface of SnS_2_. The thicknesses of SnS_2_ were all above 10 Å, and a vacuum layer of 15 Å was set up. During the calculation, the top 3 layers of atoms were in the relaxation state and the bottom layer was fixed. The parameters were set as follows: the truncation energy was 650 eV when the convergence accuracy of the self-consistent cycle was 1.0 × 10^−6^ eV/atom, the interatomic stress error was less than 0.05 GPa, the maximum interatomic displacement convergence criterion was set to 1.0 × 10^−3^ Å, the total energy convergence of the system was controlled at the criterion of 1.0 × 10^−5^ eV/atom, and the system reached equilibrium when the above conditions were satisfied.

## 3. Results and Discussions

### 3.1. Structure and Morphology Differences of the SnS_2_

**Figure 1:**
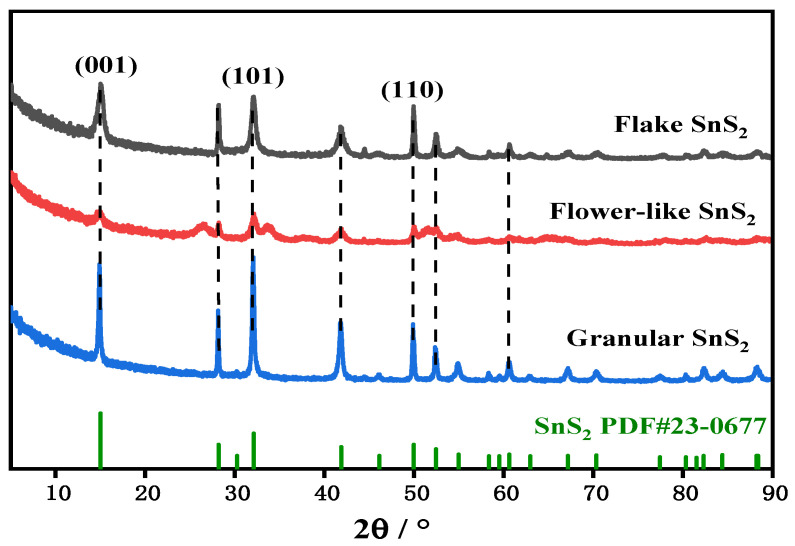
XRD patterns of SnS_2_ with different morphologies.

The crystal structures of SnS_2_ with different morphologies were analyzed through XRD, and the results are shown in Figure 1. Among them, the peaks corresponding to crystal faces (001), (101), and (110) were located at 15.0°, 32.1°, and 50.0°, respectively [32,33]. The peaks of all samples matched exactly with the standard card (PDF#23-0677) of hexagonal crystal system SnS_2_, confirming the integrity of the crystal structure; meanwhile, no impurity peaks (e.g., SnO_2_ or monomorphic S) were observed, indicating that the SnS_2_ was successfully prepared. Although the peaks of the three samples have similar positions on each crystal surface, the shapes of the peaks have large differences, which could be observed under the SEM.

**Figure 2:**
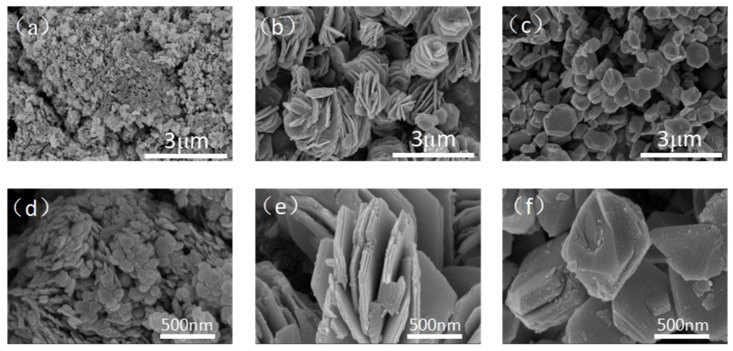
SEM images of SnS_2_ with different morphologies. (**a**) Flake-like SnS_2_. (**b**) Flower-like SnS_2_. (**c**) Granular SnS_2_. (**d**) Flake-like SnS_2_. (**e**) Flower-like SnS_2_. (**f**) Granular SnS_2_. Scale bars: 3 µm for (**a**–**c**); 500 nm for (**d**–**f**).

The morphology of the three SnS_2_ samples was analyzed through SEM, as shown in Figure 2. The SnS_2_ samples demonstrated pronounced distinctions in both morphological characteristics and particle dimensions. For the flake SnS_2_ (Figure 2a), it mainly showed uniform flakes with a size of about 200 nm and a thickness of about 20 nm and no tendency of agglomeration between the flakes. For the flower-like SnS_2_ (Figure 2b), it is mainly assembled by relatively thicker and larger SnS_2_ flakes in an undirected manner, with relative independence flower to flower, a wide size distribution of about 1~3 μm, and a thickness of its SnS_2_ flakes of about 100 nm. For granular SnS_2_, the surface is very smooth and dense, the outline is sharp, and the particle size distributed at about 0.5~1 μm. From the above, it can be seen that the prepared SnS_2_ samples have obvious differences in morphology and structure, and the morphology and surface area of the SnS_2_ sample may have a great influence on the adsorption process of MB.

The specific surface areas and pore structures of the samples were analyzed using a surface area and porosity analyzer, and the results are summarized in Table 1. Among the three samples, flower-like SnS_2_ exhibited the highest specific surface area of 31.70 m^2^/g, indicating the presence of abundant exposed active sites and hierarchical porous structures. Such characteristics are favorable for adsorption processes and can effectively enhance the interaction between adsorbent and adsorbate molecules. In comparison, flake SnS_2_ and granular SnS_2_ showed relatively lower specific surface areas of 18.45 m^2^/g and 19.85 m^2^/g, respectively. Although the granular structure possessed smaller particle sizes, its surface area remained lower than that of flower-like SnS_2_, suggesting that the self-assembled flower-like architecture contributes more significantly to surface exposure and pore accessibility.

Furthermore, the BJH pore size distribution analysis revealed that flower-like SnS_2_ possessed a broader mesoporous distribution with an average pore diameter of approximately 9.70 nm. The interconnected mesoporous channels can facilitate mass transfer and provide more accessible adsorption sites for methylene blue molecules. In contrast, flake SnS_2_ exhibited larger pore diameters but fewer accessible pores, while granular SnS_2_ showed intermediate characteristics.

These results demonstrate that the morphology of SnS_2_ plays a crucial role in determining both specific surface area and pore structure, which subsequently influence adsorption performance.

### 3.2. Effect of SnS_2_ Morphology on the Adsorption of MB at Different pH

**Figure 3:**
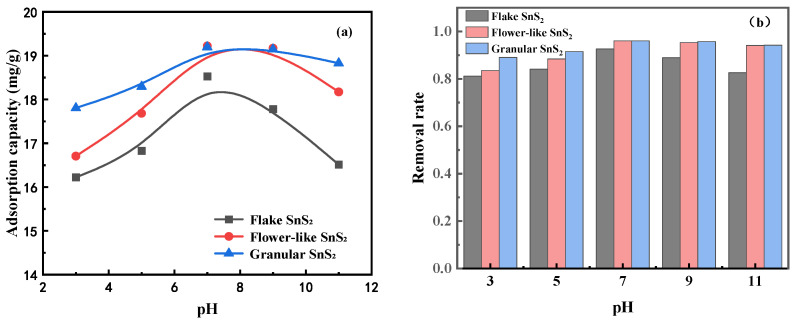
(**a**) Adsorption capacity of MB on SnS_2_ at different pH values; (**b**) removal rate of MB on SnS_2_ at different pH values.

To investigate the effect of SnS_2_ morphology on MB adsorption at different pH values, experiments were carried out under fixed conditions. At room temperature, MB solution of 10 mg/L, dosage of SnS_2_ 0.5 g/L, stirring speed of 600 r/min, and adsorption time of 240 min, the effect of solution pH on the MB adsorption of different SnS_2_ is shown in Figure 3. As can be seen, the adsorption of MB on each SnS_2_ at different pH values showed a similar trend; with the increase in pH, the adsorption capacity first increases and then decreases, and the adsorption amount under alkaline conditions is higher than that under acidic conditions. At pH < 7, the adsorption capacity of all SnS_2_ showed an increasing trend, the adsorption capacity of granular SnS_2_ was bigger than that of flower-like SnS_2_, and the adsorption capacity of flake SnS_2_ was the smallest. The maximum adsorption of each SnS_2_ was obtained at pH = 7, with maximum adsorption of 19.2 mg/g for granular SnS_2_ and 19.2 mg/g for flower-like SnS_2_, for which the removal rates are 96% and 18.5 mg/g. For flake SnS_2_, the removal rate is 92%. At pH > 7, every sample exhibits a decreasing trend of adsorption and removal rate. It can be seen that the morphology of SnS_2_ and pH made a great difference in the effect on MB adsorption.

**Figure 4:**
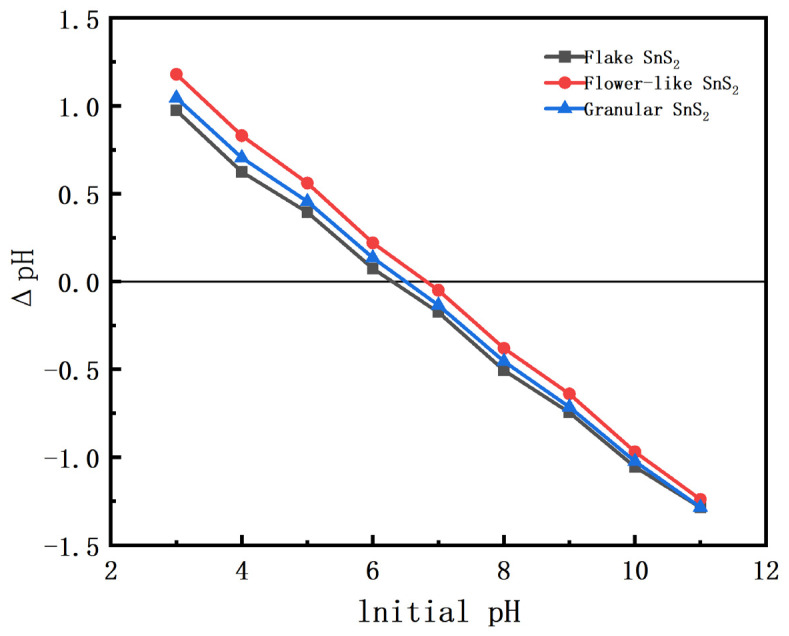
Determination of pH_pzc_ of SnS_2_ with different morphologies using the pH drift method.

The point of zero charge (pH_pzc_) of SnS_2_ samples with different morphologies was determined using the pH drift method. The initial pH of the solution was adjusted from 3 to 11, and the final pH after equilibrium was recorded. As shown in Figure 4, the pH_pzc_ values of flake, flower-like, and granular SnS_2_ were determined to be approximately 6.3, 6.8, and 6.5, respectively. When the solution pH is higher than the pH_pzc_, the surface of SnS_2_ becomes negatively charged, which enhances the electrostatic attraction toward cationic methylene blue molecules. This result explains why the adsorption capacity reaches its maximum near neutral conditions (pH ≈ 7), as observed in the adsorption experiments.

### 3.3. Effect of SnS_2_ Morphology on the Adsorption Kinetics of MB

**Figure 5:**
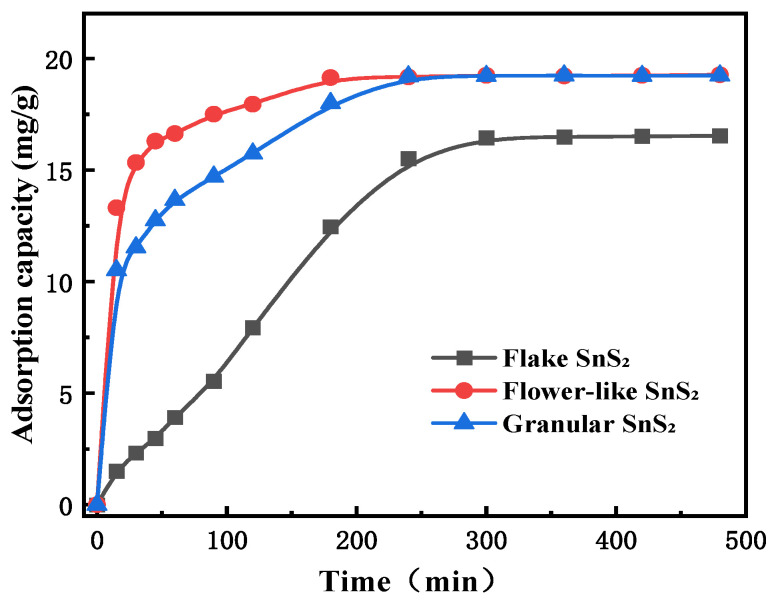
The adsorption capacity of SnS_2_ under different times.

Subsequently, the effect of adsorption time on the adsorption capacity of different shapes of SnS_2_ was investigated to better understand the adsorption kinetics. Figure 5 shows the effect of adsorption time on the adsorption capacity of different shapes of SnS_2_ under the conditions of MB solution 10 mg/L, adsorbent dosage 0.5 g/L, pH 7, and stirring speed 600 r/min. It can be seen that with increasing adsorption time, the adsorption capacity of each SnS_2_ showed a trend of rapid increase and then tended to equilibrium. In the first 200 min, the adsorption rate of MB on SnS_2_ was flower-like SnS_2_ > granular SnS_2_ > flake SnS_2_. For flower-like SnS_2_, with the increase in time, its adsorption capacity gradually increased from 13.3 mg/g to 240 min, began to level off, and reached the maximum adsorption capacity of 19.3 mg/g at 480 min. For granular SnS_2_, with the increase in time, its adsorption capacity gradually increased from 10.5 mg/g to 240 min, began to level off, and reached the maximum of 19.3 mg/g at 480 min. And, for flake SnS_2_, the adsorption capacity gradually increased from 1.5 mg/g to 240 min with increasing time and then leveled off at 480 min and reached its maximum 16.5 mg/g. The adsorption capacity of granular SnS_2_ was similar to that of flower-like SnS_2_ in the growth period, while both were higher than that of flake SnS_2_.

In order to further evaluate the adsorption of MB on the surface of SnS_2_ with different morphologies, the adsorption data were fitted using the pseudo-first-order kinetic model and pseudo-second-order kinetic model, as shown in Equations (2) and (3), respectively.

(2) $$\ln(q_{e}-q_{t})=\ln q_{e}-k_{1}t$$

(3) $$\frac{t}{q_{t}}=\frac{1}{k_{2}q_{e}^{2}}+\frac{1}{q_{e}}t$$

In these equations, *q_e_* is the equilibrium adsorption capacity, mg·g^−1^; *q_t_* is the adsorption capacity at time *t*, mg·g^−1^; *k*_1_ is the rate constant fitted to the pseudo-first-order kinetic model, min^−1^; and *k*_2_ is the rate constant fitted to the pseudo-second-order kinetic model, g·mg^−1^·min^−1^.

**Figure 6:**
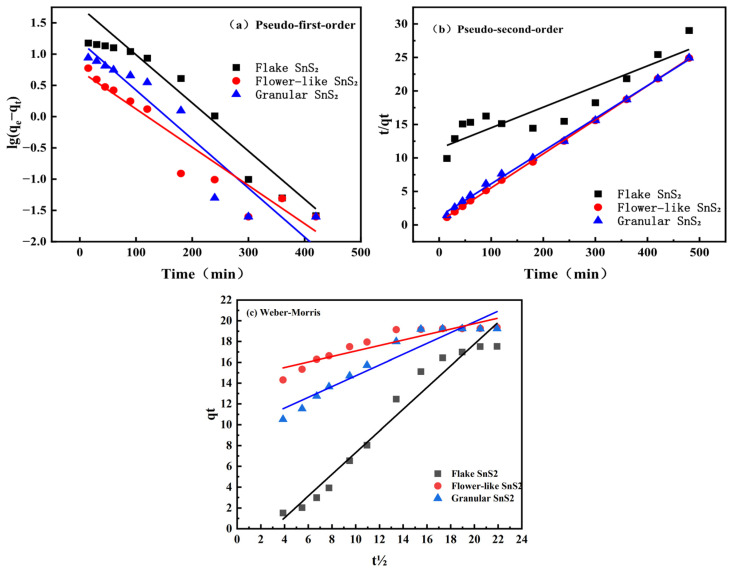
Kinetic model fittings for methylene blue (MB) adsorption onto SnS_2_ with different morphologies: (**a**) pseudo-first-order model, (**b**) pseudo-second-order model, and (**c**) Weber–Morris intraparticle diffusion model.

The fitting results are shown in Figure 6 and Table 2. From the correlation coefficients, the fitted pseudo-first-order kinetic model for the flake SnS_2_ is 0.941, while the fitted pseudo-second-order kinetic model is 0.829. We fitted flake SnS_2_ with the Weber–Morris model, and the fitting coefficient 0.960 and its adsorption rate slowed down, limiting intraparticle diffusion. The intraparticle diffusion model (Weber–Morris model) was applicable. The correlation coefficients of the fitted pseudo-second-order kinetic model are 0.999 for the flower-like SnS_2_ and 0.998 for the granular SnS_2_. The correlation coefficients of 0.999 for flower-like SnS_2_ and 0.998 for granular SnS_2_ are larger than those of the pseudo-first-order kinetic model, so they are more consistent with the pseudo-second-order kinetic model. The 2D flake structures tend to stack and agglomerate in the aqueous solution. This stacking creates narrow, restricted channels that heavily limit the intraparticle diffusion of the relatively large MB molecules.

### 3.4. Effect of SnS_2_ Morphology on the Adsorption Isotherm of MB

**Figure 7:**
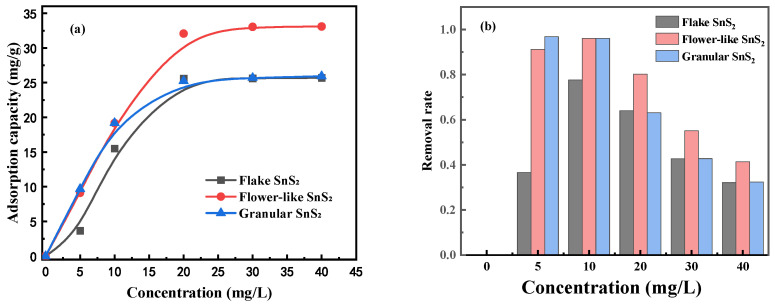
(**a**) The effect of initial MB concentration on the SnS_2_ for MB adsorption. (**b**) The removal rate of MB by SnS_2_ at different concentrations of MB.

The effect of SnS_2_ morphology on MB adsorption isotherm was studied under room temperature, adsorbent 25 mg, pH 7, adsorption time 240 min, and stirring speed 600 r/min. The effect of the initial concentration of MB on the adsorption capacity is shown in Figure 7. The adsorption capacity of all SnS_2_ initially increased and then leveled off, and the removal rate of SnS_2_ on MB increased first and then decreased. In the range of initial MB solution concentrations from 5 to 10 mg/L, the adsorption capacity of flower-like SnS_2_ and granular SnS_2_ was quite similar, both increasing from 9 mg/g to 19 mg/g, and the removal rate of MB reached 96%. In the range of 10 to 20 mg/L, the growth rate of flower-like SnS_2_ was significantly higher than that of granular SnS_2_, with the adsorption capacity of flower-like SnS_2_ increasing from 19 mg/g to 32.1 mg/g, and the removal rate dropped to 95.3% while the adsorption capacity of granular SnS_2_ increased to 25.2 mg/g and the removal rate dropped to 63.1%. After 20 mg/L, both flower-like SnS_2_ and granular SnS_2_ tended to level off. Compared to flower-like SnS_2_, flake SnS_2_ had a smaller adsorption capacity, increasing from 3.7 mg/g at 5 mg/L to 25.6 mg/g at 20 mg/L and then leveling off to reach an adsorption capacity similar to that of granular SnS_2_.

The Langmuir and Freundlich adsorption isotherm models were used to analyze the adsorption process of MB adsorption on SnS_2_. The equations for the two adsorption isotherm models are as follows:

(4) $$\frac{C_{e}}{q_{e}}=\frac{1}{bq_{max}}+\frac{C_{e}}{q_{max}}$$

(5) $$R_{L}=\frac{1}{1+bC_{0}}$$

(6) $$lnq_{e}=lnK_{F}+\frac{lnC_{e}}{n}$$

In these equations, *C*_0_ is the initial MB concentration, mg/L; *C_e_* is the concentration of MB at equilibrium, mg/L; *q_e_* is the equilibrium adsorption capacity, mg/g; *q_max_* is the maximum adsorption capacity, mg/g; *b* is the Langmuir constant; *R_L_* is the separation factor that characterizes adsorption performance; *K_F_* is the constant in the isotherm equation; and n is a constant related to temperature.

**Figure 8:**
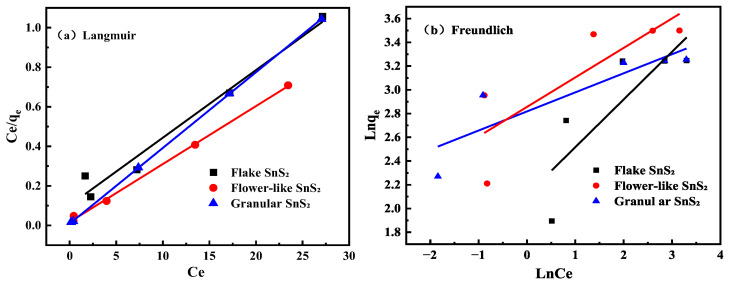
Adsorption isotherm fittings for methylene blue (MB) onto SnS_2_ with different morphologies: (**a**) Langmuir model and (**b**) Freundlich model.

The fitting results of the experimental data are shown in Figure 8 and Table 3. It can be seen that the correlation coefficient of the Langmuir adsorption isotherm model for flake SnS_2_ is 0.965, flower-like SnS_2_ is 0.998, and granular SnS_2_ is 0.999. All of the values are greater than that of the Freundlich adsorption isotherm model, indicating that adsorption processes of MB on different morphologies of SnS_2_ are more consistent with the Langmuir adsorption isotherm model. This demonstrates that the adsorption process of MB on different morphologies SnS_2_ belongs to monolayer adsorption.

To evaluate the adsorption performance of SnS_2_, a comparison with previously reported adsorbents is presented in Table 4 [34,35,36,37]. It can be observed that the adsorption capacity of flower-like SnS_2_ (33.1 mg/g) is higher than that of many conventional adsorbents, such as activated carbon, and comparable to other SnS_2_-based nanostructures. Compared with MoS_2_ and WS_2_, SnS_2_ shows competitive adsorption performance, which can be attributed to its intrinsic hydrophilicity and favorable surface properties. Furthermore, the enhanced performance of flower-like SnS_2_ indicates that morphology engineering is an effective strategy to improve adsorption efficiency. These results demonstrate that the prepared SnS_2_, especially the flower-like structure, is a promising adsorbent for dye removal in aqueous systems.

### 3.5. Effect of SnS_2_ Morphology on the Adsorption Thermodynamics of MB

**Figure 9:**
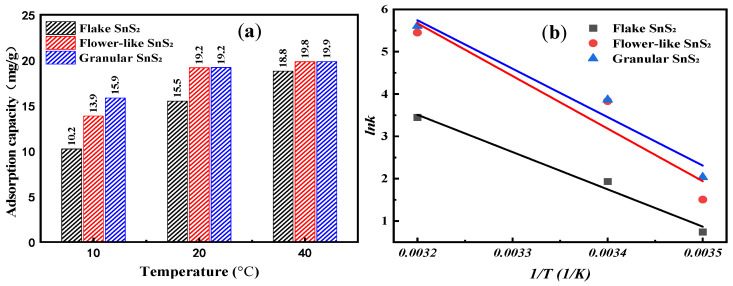
(**a**) The effect of temperature on MB adsorption on SnS_2_; (**b**) the fitting of MB adsorption thermodynamics on SnS_2_.

Next, in order to evaluate the effect of SnS_2_ morphology on the thermodynamics of MB adsorption, experiments were carried out at different temperatures. Other parameters remained the same. Under MB concentration 10 mg/L, adsorbent dosage 0.5 g/L, adsorption time 240 min, pH 7, and stirring speed 600 r/min, the effect of temperature on the equilibrium adsorption capacity is shown in Figure 9a. When the temperature ranges from 10 °C to 40 °C, the equilibrium adsorption capacity of flake SnS_2_, flower-like SnS_2_, and granular SnS_2_ gradually increases with rising temperature. This may be due to the temperature promoting molecular diffusion and the activity of surface-active sites. When the temperature reaches 40 °C, the equilibrium adsorption capacities of all SnS_2_ reach their maximum values, with the maximum equilibrium adsorption capacity of flower-like SnS_2_ 19.8 mg/g and granular SnS_2_ 19.9 mg/g, which are greater than the maximum adsorption capacity of flake SnS_2_ 18.8 mg/g.

To explore the thermodynamics of different morphologies of SnS_2_ in MB adsorption, the experimental data were fitted using an adsorption thermodynamic equation. The calculation equation for the thermodynamic equilibrium constant K_d_ is as follows:

(7) $$K_{d}=\frac{q_{e}}{c_{e}}\cdot \frac{m}{V}$$

The equation for the thermodynamic parameters Δ*G^θ^*, Δ*S^θ^,* and Δ*H^θ^* are as follows:

(8) $$∆G^{\theta }=-RT\ln K_{d}$$

(9) $$\ln K_{d}=\frac{-∆G^{\theta }}{RT}=\frac{-∆H^{\theta }}{RT}+\frac{∆S^{\theta }}{R}$$

where *T* is the thermodynamic temperature of adsorption, K. *R* is the ideal gas constant, J·mol^−1^·K^−1^. *m* is the mass of the adsorbent, g. *V* is the volume of the adsorbate solution, L. By using this equation, *K_d_* becomes dimensionless [38].

Using Equation (9) to fit the experimental data of the graph and calculate the relevant parameters, the fitting and calculation results are shown in Figure 9b and Table 5, respectively. From the contents, it can be seen that at temperature 283 K, Δ*G^θ^* for flake SnS_2_ is −0.115 KJ·mol^−1^, for flower-like SnS_2_ is −1.912 KJ·mol^−1^, and for granular SnS_2_ is −3.156 KJ·mol^−1^. At temperature 293 K, Δ*G^θ^* for flake SnS_2_ is −3.021 KJ·mol^−1^, for flower-like SnS_2_ is −7.648 KJ·mol^−1^, and for granular SnS_2_ is −7.729 KJ·mol^−1^. At a temperature of 313 K, Δ*G^θ^* for flake SnS_2_ is −7.170 KJ·mol^−1^, for flower-like SnS_2_ is −12.385 KJ·mol^−1^, and for granular SnS_2_ is −12.784 KJ·mol^−1^. Therefore, Δ*G^θ^* for the adsorption of MB by SnS_2_ with different morphologies are all negative, indicating that the adsorption process is spontaneous. As the temperature increases, Δ*G^θ^* decrease, suggesting that raising the temperature is favorable for adsorption. In addition, both the values of Δ*H^θ^* and the Δ*S^θ^* for MB adsorption on SnS_2_ performed in the order of flower-like SnS_2_ > granular SnS_2_ > flake SnS_2_, suggesting that MB is more easily absorbed on the flower-like SnS_2_ than granular SnS_2_ and final flake SnS_2_.

### 3.6. Mechanism Difference of MB Adsorption on Various Morphologies SnS_2_

**Figure 10:**
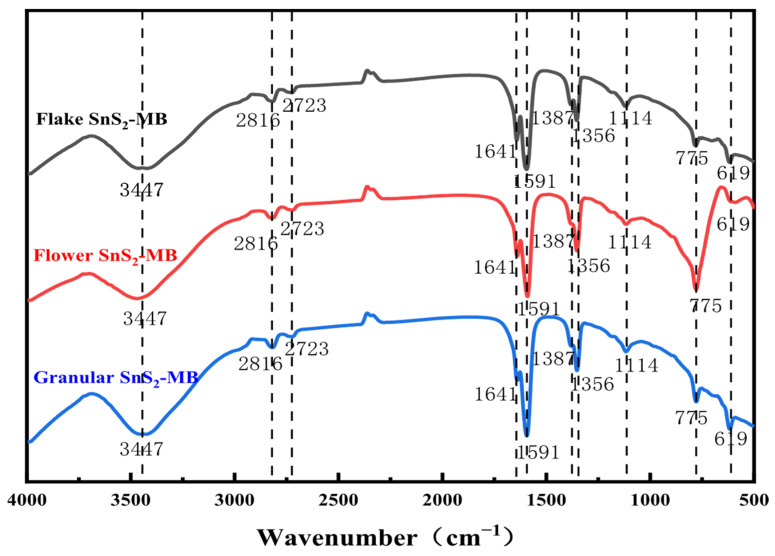
FT-IR of different morphologies of SnS_2_ after MB adsorption.

Finally, in order to further elucidate the adsorption mechanism, Fourier transform infrared spectroscopy was performed on the SnS_2_ samples after adsorption of MB. FT-IR was used to analyze SnS_2_ after MB adsorption, and the results are shown in Figure 10. Regardless of whether the SnS_2_ is in a flake, flower-like, or granular form, many significant absorption peaks were observed at some same positions after the adsorption of MB. The broad absorption peak at 3345 cm^−1^ corresponds to the stretching vibration of –OH, the peak at 2702 cm^−1^ corresponds to the stretching vibration of the –CH_3_, and the peak at 1654 cm^−1^ is attributed to the key characteristic peaks of the MB molecule, specifically the stretching vibration of C=N in the aromatic and heterocyclic structures. The peaks at 1594 cm^−1^ and 1389 cm^−1^ are attributed to the stretching vibrations of C=C and C–H, respectively, while the peak at 1139 cm^−1^ reflects the bending vibration of C–H in the heterocycle [39]. These results confirm the successful adsorption of MB on the surface of SnS_2_.

**Figure 11:**
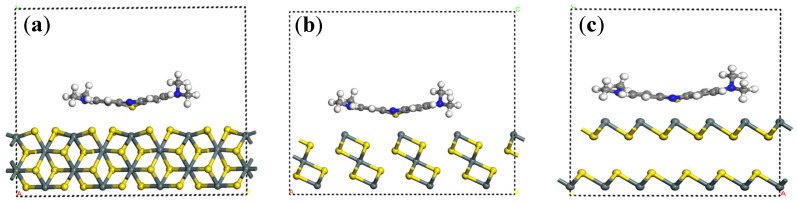
Models of MB adsorbed on different morphologies of SnS_2_: (**a**) flake SnS_2_, (**b**) flower-like SnS_2_, (**c**) granular SnS_2_.

To investigate the mechanism of the differences in MB adsorption on the surfaces of SnS_2_ with different morphologies, DFT simulations were conducted to simulate the adsorption process of the MB molecule on the surfaces of different SnS_2_ morphologies, with their adsorption structures shown in Figure 11. The DFT figures are established through the adsorption of MB molecules on the different crystal surfaces of SnS_2_. The crystal surfaces were built based on the different morphologies of SnS_2_ and the flower-like, flake, and granular morphologies are pointed out corresponding to the crystal surfaces (110), (101), and (001), respectively [29,31]. The MB molecules seem to be sitting on the top of the crystal surfaces according to the calculation results of the DFT simulation. Their adsorption energies were very different and are listed in the Table 6, which indicates their interactions.

In order to study the mechanism of the adsorption differences of MB on SnS_2_ with different morphologies, the adsorption energies of MB on the surfaces of SnS_2_ were calculated through DFT. The adsorption energy was calculated using Equation (10).

(10) $$\Delta E_{Adsorbent/MB}=E_{Adsorbent/MB}-E_{Adsorbent}{-E}_{MB}$$

Adsorbent represents the different morphology SnS_2_, Δ*_Adsorbent/MB_* denotes the adsorption energy between SnS_2_ and MB, and *E_Adsorbent/MB_* and *E_Adsorbent_* represent the energy of the adsorbent–MB system and the adsorbent system, respectively.

The results of the adsorption energies are show in Table 6. Before the adsorption of MB, the surface energies of flake SnS_2_, flower-like SnS_2_, and granular SnS_2_ were −51,173.330 eV, −38,463.866 eV, and −28,909.948 eV, respectively. After the adsorption of MB, the surface energies of flake SnS_2_, flower-like SnS_2_, and granular SnS_2_ were −52,355.652 eV, −39,646.141 eV, and −30,092.254 eV, respectively. Compared to before adsorption, their differences were −0.222 eV, −0.175 eV, and −0.206 eV, respectively. The larger the difference, the stronger the adsorption capacity. Therefore, it can be concluded that the adsorption energy of MB on different morphologies of SnS_2_ is flower-like > granular > flake SnS_2_. The big difference in adsorption energy of MB on SnS_2_ surfaces with different morphologies may be the main reason for their different adsorption properties.

### 3.7. Reusability

**Figure 12:**
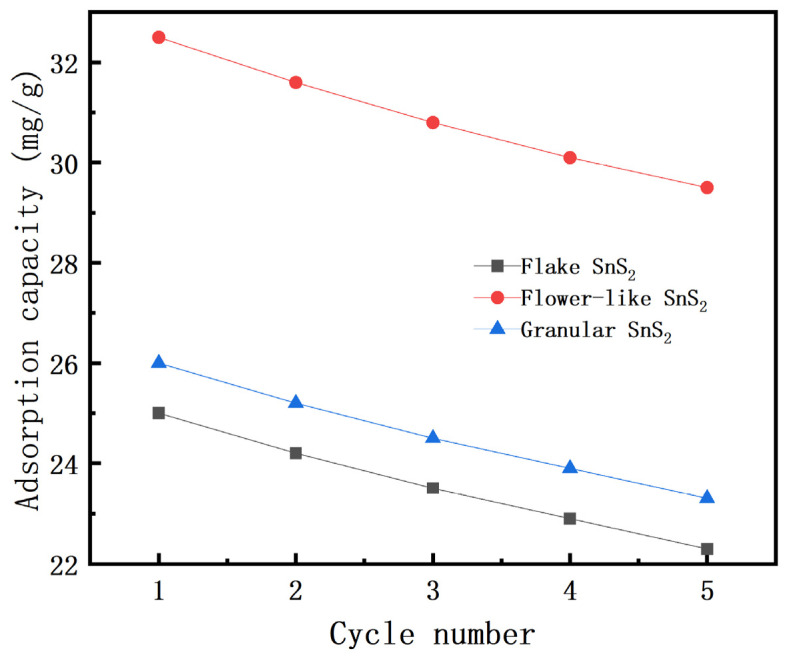
Cycle performance of SnS_2_ with different morphologies toward methylene blue adsorption.

The reusability of SnS_2_ was evaluated over five adsorption–desorption cycles. As shown in Figure 12, the adsorption capacity of flower-like SnS_2_ decreased from 32.5 mg/g in the first cycle to 29.5 mg/g after five cycles, retaining approximately 90.8% of its initial performance.

Similarly, flake and granular SnS_2_ retained about 89.2% and 89.6%, respectively. The slight decrease in adsorption capacity may be attributed to incomplete desorption of MB molecules and partial blockage of active sites.

These results indicate that SnS_2_ exhibits good structural stability and reusability, demonstrating its potential for practical wastewater treatment applications.

## 4. Conclusions

In this study, three different morphologies of SnS_2_ (flake, flower-like, and granular) were successfully synthesized using a one-step hydrothermal method by precisely regulating the microscopic conditions of the synthesis reaction. The experimental data showed that the adsorption performance of flower-like SnS_2_ for MB was significantly better than that of the other two forms, and the excellent performance and high specific surface area provided a larger effective contact area (BET specific surface area of up to 31.7 m^2^/g) and a rich variety of active sites, which created a favorable interfacial environment for the adsorption process. Thermodynamic studies showed that all SnS_2_ adsorption on MB was a spontaneous process (Δ*G* < 0), and the warming up contributed to the enhancement of the adsorption capacity, a phenomenon that was highly consistent with Langmuir’s model of monomolecular layer adsorption. Notably, the density functional theory (DFT) calculations revealed that the adsorption energies between SnS_2_ surfaces and MB molecules with different morphologies were significantly different, which might be the essential reason for the difference in adsorption performance. This study not only reveals the mechanism of nanomaterial shape engineering on the regulation of adsorption performance but also provides important structure–property relationship guidance for the design of efficient adsorbents.

## Figures and Tables

**Table 1:** Specific surface areas of SnS_2_ samples with different morphologies.

Sample	SSA (m^2^/g)	D_p_ (nm)
flake SnS_2_	18.45	32.27
flower-like SnS_2_	31.70	9.70
granular SnS_2_	19.85	19.34

**Table 2:** Parameters of the fitting for adsorption kinetics of MB on SnS_2_.

Sample	Pseudo-First-Order			Pseudo-Second-Order			Weber–Morris	
	*q_e_*/mg·g^−1^	*k*_1_	*R* ^2^	*q_e_*/mg·g^−1^	*k*_2_	*R* ^2^	*k_p_*	*R* ^2^
Flake SnS_2_	16.538	0.008	0.941	16.538	0.001	0.829	1.040	0.960
Flower-like SnS_2_	19.269	0.007	0.928	19.269	0.006	0.999	0.264	0.831
Granular SnS_2_	19.245	0.009	0.902	19.245	0.002	0.998	0.518	0.900

**Table 3:** Parameters of the fitting for adsorption isothermal of MB on SnS_2_.

Sample	Langmuir Model			Freundlich Model		
	*q_max_*	*R_L_*	*R* ^2^	1/*n*	*K_F_*	*R* ^2^
Flake SnS_2_	25.7	0.061	0.965	0.402	8.295	0.696
Flower-like SnS_2_	33.1	0.014	0.998	0.249	17.393	0.694
Granular SnS_2_	25.9	0.006	0.999	0.161	16.743	0.774

**Table 4:** Comparison of adsorption capacity of SnS_2_ and related 2D materials for MB removal.

Material Morphology	*q_max_* (mg/g)	Conditions (pH, T)
Flake SnS_2_	25.7	pH = 7, 298 K
Flower-like SnS_2_	33.1	pH = 7, 298 K
Granular SnS_2_	25.9	pH = 7, 298 K
MoS_2_	22.3	pH = 7, 298 K
WS_2_	18.7	pH = 7, 298 K

**Table 5:** Fitting parameters of MB adsorption thermodynamics on SnS_2_.

Sample	T (K)	Δ*G^θ^* (KJ·mol^−1^)	Δ*H^θ^* (KJ·mol^−1^)	Δ*S^θ^* (J·mol^−1^)
Flake SnS_2_	283	−0.115	73.283	257.925
	293	−3.021		
	313	−7.170		
Flower-like SnS_2_	283	−1.912	103.358	372.116
	293	−7.648		
	313	−12.385		
Granular SnS_2_	283	−3.156	95.166	346.515
	293	−7.729		
	313	−12.784		

**Table 6:** Surface energy calculation results.

Sample	*E_Asorbent/MB_*	*E_Asorbent_*	*E_MB_*	Δ*E_Asorbent/MB_*
Flake SnS_2_	−52,355.652	−51,173.330	−1182.100	−0.222
Flower-like SnS_2_	−39,646.141	−38,463.866	−1182.100	−0.175
Granular SnS_2_	−30,092.254	−28,909.948	−1182.100	−0.206

## Data Availability

The original contributions presented in this study are included in the article. Further inquiries can be directed to the corresponding author.
